# Genomic analysis of *Escherichia coli* strains isolated from diseased chicken in the Czech Republic

**DOI:** 10.1186/s12917-020-02407-2

**Published:** 2020-06-10

**Authors:** Aneta Papouskova, Martina Masarikova, Adam Valcek, David Senk, Darina Cejkova, Eva Jahodarova, Alois Cizek

**Affiliations:** 1 Department of Infectious Diseases and Microbiology, Faculty of Veterinary Medicine, University of Veterinary and Pharmaceutical Sciences Brno, Brno, Czech Republic; 2 Central European Institute of Technology, University of Veterinary and Pharmaceutical Sciences Brno, Brno, Czech Republic; 3 Department of Biology and Wildlife Diseases, Faculty of Veterinary Hygiene and Ecology, University of Veterinary and Pharmaceutical Sciences Brno, Brno, Czech Republic; 4 Department of Immunology, Veterinary Research Institute, Brno, Czech Republic

**Keywords:** Avian pathogenic *E. coli*, Extraintestinal pathogenic *E. coli*, Virulence-associated genes, Avian colibacillosis, Whole-genome sequencing

## Abstract

**Background:**

Avian pathogenic *Escherichia coli* (APEC) can cause various extraintestinal infections in poultry, resulting in massive economic losses in poultry industry. In addition, some avian *E. coli* strains may have zoonotic potential, making poultry a possible source of infection for humans. Due to its extreme genetic diversity, this pathotype remains poorly defined. This study aimed to investigate the diversity of colibacillosis-associated *E. coli* isolates from Central European countries with a focus on the Czech Republic.

**Results:**

Of 95 clinical isolates subjected to preliminary characterization, 32 were selected for whole-genome sequencing. A multi resistant phenotype was detected in a majority of the sequenced strains with the predominant resistance to β-lactams and quinolones being associated with TEM-type beta-lactamase genes and chromosomal *gyrA* mutations respectively. The phylogenetic analysis confirmed a great diversity of isolates, that were derived from nearly all phylogenetic groups, with predominace of B2, B1 and C phylogroups. Clusters of closely related isolates within ST23 (phylogroup C) and ST429 (phylogroup B2) indicated a possible local spread of these clones. Besides, the ST429 cluster carried *bla*_CMY-2, − 59_ genes for AmpC beta-lactamase and isolates of both clusters were generally well-equipped with virulence-associated genes, with considerable differences in distribution of certain virulence-associated genes between phylogenetically distant lineages. Other important and potentially zoonotic APEC STs were detected, incl. ST117, ST354 and ST95, showing several molecular features typical for human ExPEC.

**Conclusions:**

The results support the concept of local spread of virulent APEC clones, as well as of zoonotic potential of specific poultry-associated lineages, and highlight the need to investigate the possible source of these pathogenic strains.

## Background

Avian colibacillosis is a complex of several localized or systemic syndromes, affecting poultry of all age and production categories. It comprises yolk sac infection and omphalitis, leading to increased mortality rates in newly hatched chicks, cellulitis in broilers or reproductive tract infections in laying hens. Other forms of manifestation include swollen head syndrome (SHS), respiratory infections and septicemia which frequently result in death or chronic forms of infection. Avian colibacillosis thus represents a great economic burden for the poultry industry [1]. Despite its importance as a significant cause of disease, the pathogenesis of these infections is not utterly understood. For a long time APEC strains were considered merely opportunistic pathogens, predominantly, but not exclusively associated with O1, O2, O8, O78 and several other serogroups [2]. It has been demonstrated that disease-associated *E. coli* strains encode multiple putative virulence genes and significantly differ from commensals, particularly in the carriage of the ColV plasmid-associated genes, which are considered markers of poultry-adapted pathogenic strains [3–5].

The ability to cause colibacillosis in chicken defines the APEC (avian-pathogenic *E. coli*) pathotype. However, not every strain isolated from diseased chicken carries typical virulence-associated genes, underlining an opportunistic character of some types of *E. coli* infections [6]. On the other hand, APEC-like strains (carrying APEC-associated virulence traits) can be found also in the gut of healthy chicken [7, 8]. It has been suggested by Maturana et al. [9] that the APEC population composes of distinct subpathotypes associated with different syndromes, similar to the human extraintestinal pathogenic *E. coli* (ExPEC). Interestingly, the authors showed that SHS and omphalitis isolates formed two distinct groups differing in virulence, suggesting primary and opportunistic character of those infections, respectively. Similarly, chronic salpingitis-peritonitis syndrome resulting from an ascending infection and an acute peritonitis without salpingitis, probably originating from respiratory infection or gut translocation after a stress insult, can be distinguished in layers [10–12].

There is a close genetic relationship between APEC and human ExPEC. Zoonotic potential of poultry strains has been implicated. ExPEC are the main cause of urinary-tract infections (as so called uropathogenic *E. coli*, UPEC) in humans and meningitis in neonates (neonatal-meningitis *E. coli*, NMEC), and are also associated with bacteremia, sepsis, cellulitis and other potentially fatal infections [13]. Similar to APEC, these strains are characterized by the presence of various virulence-associated genes, participating in adhesion and colonization of different tissues, invasion of internal organs, iron acquisition and avoiding host’s immune responses. ExPEC are typically associated with the phylogenetic groups B2 and D, in contrast to commensal and intestinal pathogenic strains derived from groups A and B1 [14] and to APEC, which are highly variable in distribution to various phylogenetic groups [7]. Although there is no specific set of genes to define the subpathotypes [15, 16], APEC, UPEC and NMEC generally form genetically distinct groups. There is, however, a substantial overlap especially within the B2 phylogenetic group, which comprises strains isolated from both humans and chickens, showing high virulence in the chicken infection model and in the neonatal rat meningitis model with low or no host specifity [16–18]. Moreover, an isolate showing high virulence in the rat meningitis model has been found in faeces of a healthy chicken [5], another finding that suggests an association between poultry and poultry products as a potential source of human pathogens.

Recently, several highly virulent and resistant ExPEC lineages with worldwide distribution have emerged (e.g. ST131, ST95 etc.) [19]. Whereas some of them are associated exclusively with human infections, others are frequently isolated from diseased poultry or poultry products [20–25]. It is however difficult to assess the real importance of poultry as a source of human infections. Mechanisms of transmission of pathogenic clones through the production chain to humans are very complex and not quite elucidated, as well as the relationships between genetic „arsenal “of virulence and resistance-associated genes and pathogenesis of the disease. Whole-genome sequencing (WGS) represents a revolutionary tool to study these mechanisms in their complexity [26]. Moreover, an immense variability of APEC pathotype and differences in geographic distribution of specific clones underlines the importance of mapping the local situation. While several papers have reported occurrence of highly pathogenic APEC clones in different counries, the information for the Central Europe have been sporadic or is lacking [27].

## Results

### Samples collection and preliminary characterization

**Fig. 1:**
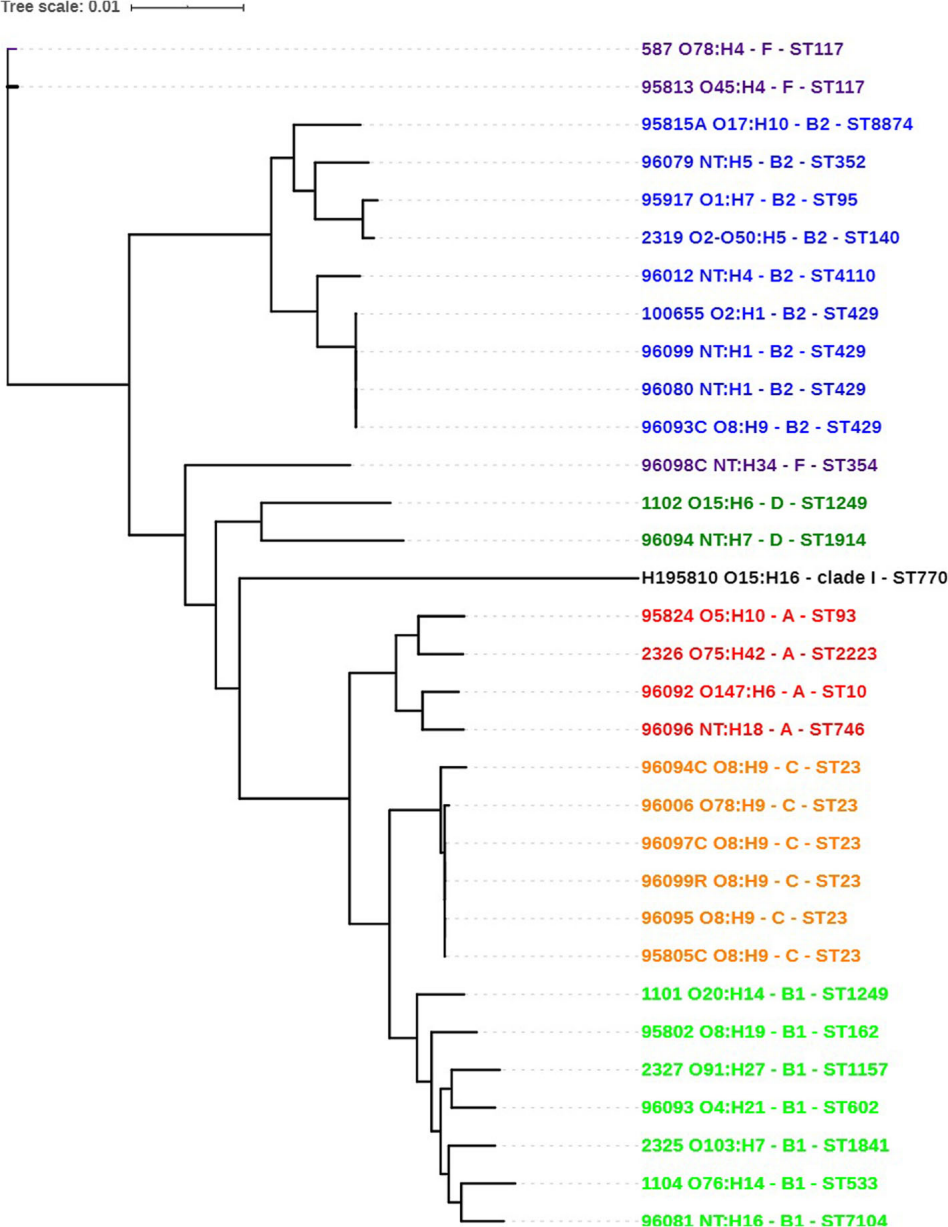
Phylogenetic analysis of sequenced isolates.  – phylogroup F;  – phylogroup B2;  – phylogroup D;  – clade I;  – phylogroup A;  – phylogroup C;  – phylogroup B1

A total of 95 isolates were subjected to preliminary characterization including serogrouping, antimicrobial resistance (AMR) testing and PCR detection of virulence and antibiotic resistance genes. The disc diffusion test showed that 69.5% were resistant to three or more groups of antimicrobials, which we considered as a criterion of multiresistance. Resistance to ampicillin was recorded in 78 isolates (82.0%), followed by resistance to nalidixic acid (62 isolates; 65.3%), sulphonamides (45; 47.4%) and sulphonamides-trimethoprim (28; 29.5%). Nineteen isolates (20.0%) showed reduced susceptibility to ciprofloxacin (additional file 1 – Figure 1). Using four antisera (O1, O8, O18 and O78), 49 isolates (52%) were typeable, with predominant serogroups O1 (30; 32%) and O8 (13; 14%). Four isolates reacted to O78 and two against O18 antisera.

The *bla*_TEM_ gene was detected in 45 isolates (47.4%). Other prevalent genes detected by PCR included *tet*(A) (26 isolates; 27.4%) and *sul2* (28.4%). As for virulence genes, most isolates carried typical APEC plasmid-associated genes *iss* (75; 78.9%), *iroN* (73; 76.8%), *iut*(A) (68; 71.6%), whereas others, plasmid- or chromosome-associated genes, e.g. *cvaC* (49; 51.6%), *frz*_*orf4*_ (44; 46.3%), *tsh* (32; 33.7%) and *felA* (7; 7.4%) were less prevalent.

### In silico serotyping, MLST and phylogenetic analysis of 32 selected isolates

We found a diversity of serogroups in the collection of 32 isolates subjected to whole genome sequencing. Overall 14 different O types and 16 H types were identified and 10 isolates failed to be typed by WGS (the results are summarized in Table 1, additional file 1). Except for serogroup O8 (7 isolates), the remaining serogroups were only represented by one or two isolates. The predominance of O8 serogroup appeared as a selection bias since only Czech isolates were selected for sequencing. Of the O8 serogroup, six isolates belonged to the O8:H9 serotype, most of them to ST23 type.

The isolates were derived from all phylogroups according to the Clermont scheme [28] except for the group E: group F (3 isolates), B2 (9 isolates), D (2 isolates), clade I (1 isolate), A (4 isolates), C (6 isolates), B1 (7 isolates).

The MLST analysis identified 22 distinct sequence types (please see Table 1 in the supplementary material and Fig. 1), most of them represented only by a single isolate (ST352, ST95, ST140, ST354, ST93, ST4110, ST1249, ST1914, ST770, ST2223, ST746, ST1249, ST162, ST1157, ST602, ST1841, ST533, ST7104). Two isolates were typed as ST117 belonging to the phylogenetic group F; ST429 of the B2 group and ST23 of the C group were detected in 4 and 6 isolates, respectively.

The core genome consisted of 2763 genes (55.28 kbp). The phylogenetic tree based on the core SNPs corresponded to the structure of *E. coli* phylogeny. Groups F, D and clade I were represented by only a few isolates and did not form any distinct clusters except the minor subcluster of the two group D isolates; two ST117 isolates from the F phylogroup were unrelated to other isolate of F phylogroup (ST354) and formed their own distinct clade. In the B2 cluster two subclusters (B2a, B2b) were found; B2b subcluster was formed by four closely related ST429 isolates and one ST4110. Another cluster included isolates from phylogroups A, C and B1. Interestingly, all isolates of the C group belonged to ST23, O8:H9 serotype (with one exception of O78:H9 serotype).

### Identification of resistance genes

*bla*_TEM-1_ (9/32; 28.1%) and a combination of *bla*_TEM-106_, _135_ (6/32; 18.8%) belonged amongst the most prevalent resistance genes. A combination of plasmid-mediated β-lactamase genes *bla*_CMY-2, − 59_ was detected in four isolates (12.5%), three of them belonging to the ST429, the remainig one to ST354. PMQR (plasmid-mediated quinolone resistance) gene *qnrS1* was carried by seven isolates (21.8%) within sequence types 23 and 429. Other identified AMR genes were *sul1* (7/32; 21.8%), *sul2* (8/32; 25.0%), *dfrA14* (1/32; 3.0%), *dfrA15* (4/32; 12.5%), *dfrA5* (1/32; 3.0%), *tet*(A) (12/32; 37.5%), *tet*(B) (2/32; 6.3%), *aadA* (6/32; 18.8%), *aac (3)-*VIa (3/32; 9.4%), *ant (2)-1a* (1/32; 3.0%), *aph (3)-1b* (6/32; 18.8%), *aph (3)-1a* (2/32; 6.3%), *aph (6)-1d* (5/32; 15.6%), *catA1* (2/32; 6.3%), *floR* (1/32; 3.0%) and *bla*_TEM-30_ (1/32; 3,0%). In addition, all isolates showed the presence of genes encoding components of various multidrug efflux pumps, participating in resistance to aminoglycosides, macrolides and fluoroquinolones. Except for *qnrS1*, which is associated with partial resistance to fluoroquinolones, no other PMQR gene was detected. Reduced susceptibility to quinolones in most isolates appeared to be due to chromosomal mutations, especially in the *gyrA* gene (21; 65.6%), to lesser extent also in *parC* (5; 15.6%) and *parE* (1; 3%). In five ST23 isolates (15.6%), a mutation in the *ampC* promoter was detected. (For overview of resistance genes, please see the Table 1, additional file 1.)

### Identification of virulence genes

**Fig. 2:**
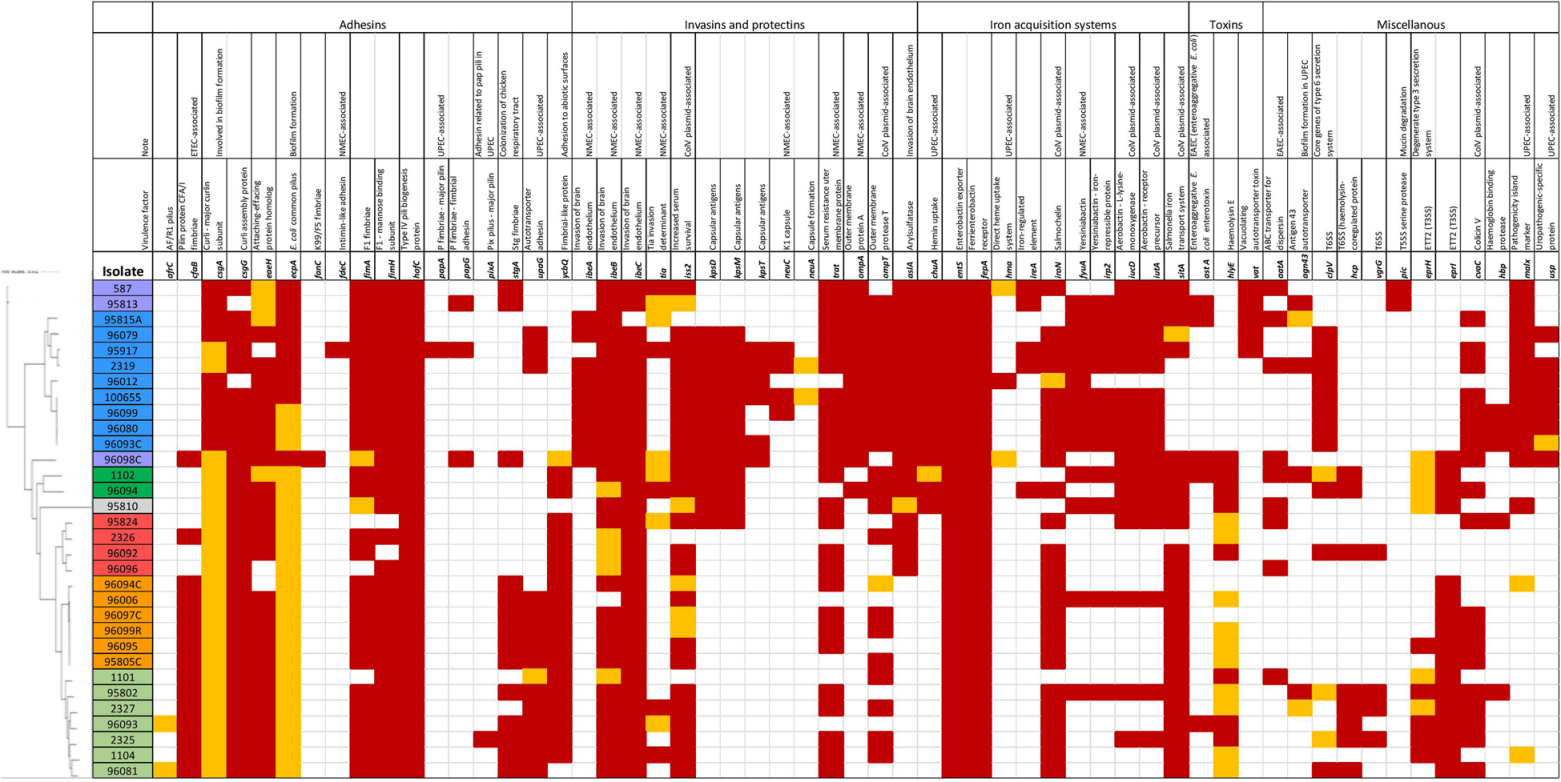
Selected virulence-associated genes in sequenced isolates.  – phylogroup F;  – phylogroup B2;  – phylogroup D;  – clade I;  – phylogroup A;  – phylogroup C;  – phylogroup B1. Red field – 100% ID; orange field - ≥95% ID

The genomic analysis confirmed a great diversity of selected isolates (see Fig. 2 and supplementary material, file 3). Overall, factors associated with adhesion and invasion, as well as siderophores were found in most isolates; more than 90% of isolates encoded F1 fimbriae, curli, *E. coli* common pilus and enterobactin. All but one isolate carried *ibeB* gene, while *ibeA* was present mostly in B2 and F phylogenetic groups, but not in isolates from other groups. A siderophore system salmochelin (81%), haemolysin F (90.6%) and serum-resistance associated proteins, Iss (87.5%) and TraT (78%) were present in most isolates with generally equal distribution in all phylogenetic groups. Full SitABCD iron transport system was detected in 78% isolates, outer membrane protease (OmpT) and colicin V synthesis protein (CvaC) in 68.8 and 59% isolates, respectively.

Several virulence- genes were associated with particular branches of the phylogenetic tree. For example, Stg fimbriae, Ycb fimbriae, CFA/1 fimbriae and genes associated with ETT2 (*E. coli* type III secretion system 2) were common in B1 and C groups, but almost or entirely absent in B2 phylogenetic group. In contrast, yersiniabactin, aerobactin and hemin receptor (*chuA*), as well as OmpA, capsular antigens (*kpsD, kpsT, kpsM*), pathogenicity-island marker *malX,* uropathopathogenic-specific protein (*usp*) and afore-mentioned brain-endothelium invasin (*ibeA*) appeared to be B2 group-associated.

Lastly, some well known virulence-associated genes were detected uniquely amongst our isolates. For example, complete *pap* operon was present only in the one ST95 isolate, as well as the intimin-like adhesin (*fdeC*). Similarly, *neuC* gene was found in ST95, ST140 and two ST429 isolates (all B2 phylogroup) and K99 (F5) fimbriae only in ST354 isolate of the F phylogroup. For overview of all virulence-associated genes detected please see the Table 2, additional file 2.

### Identification of plasmid replicons

All but one isolate harboured a replicon of the F incompatibility group, FIB replicon being the most commonly detected (31/32 isolates; 96.9%). At the same time, Col replicons were detected in most (22/32; 68.8%) isolates. Groups IncB/O/K/Z (10; 31.3%) and IncX1 (6; 18.8%) also appeared relatively frequently, while others were identified only in individual isolates. The IncHI1B replicon was identified in two isolates of the ST429 and ST23 cluster, respectively. Overall, types and number of replicons varied greatly even within the two closely related clusters. (For overview of replicons, please see the Table 1, additional file 1.)

## Discussion

The aim of current study was to evaluate diversity of colibacillosis-associated isolates from the Czech Republic. Indeed, analysis showed an immense phenotypic and genotypic variability, the isolates differing greatly in their antimicrobial-resistance phenotype, virulence genes profile and plasmid content, together having little in common. As generally acknowledged, there is no specific combination of virulence genes that would accurately define the APEC pathotype [15]. The most prevalent APEC genes are also frequently present in commensal strains. There is an abundance of adhesins and iron-transporting systems, which may be considered essential prerequsites of extraintestinal pathogenicity in all types of avian and mammalian disease, but also fitness factors enabling asymptomatic colonization of healthy hosts and effective transmission. Presence of Col-V-associated genes such as *iroN, iss, iutA, ompT* etc. is characteristic for most APEC, more than UPEC and NMEC [7], nevertheless, their exact role in pathogenesis remains unclear or controversial [29, 30]. Col-V-like plasmids are, however, acknowledged as markers of poultry-adapted pathogenic strains [5, 21].

As expected, the phylogenetic analysis also revealed a substantial diversity of isolates, that originated from all phylogenetic groups with the exception of group E, the most prevalent was B2 phylogroup, which is, along with D, considered typical group for human ExPEC [14]. However, the second most prevalent phylogroup was B1 (7 isolates), a group commonly associated with intestinal pathogenic or commensal fecal strains [31]. Interestingly, there were notable differences in virulence trait distribution among phylogenetic groups, although the isolates had been collected from the same types of infection. The idea of pathogenic strains with quite a different combination of virulence genes with alternative functions causing the same clinical disease has been proposed by Mokady et al. [32] and points out the importance of horizontal gene transfer enabling rapid adaptation to new niches by expression of certain genes in a different genetic background [33]. Notably, it was the presence of typical Col-V plasmid-associated genes such as *ompT*, *iss*, *cvaC*, *iro* and *sit* (but suprisingly not *iut*, *iuc* for aerobactin) that were equally distributed among isolates from all phylogenetic groups.

Despite the overall diversity, the phylogenetic analysis revealed two clusters (ST429, group B2, and ST23, groups C), both containing four similar isolates that were obtained from different farms in Northern Moravia. Two ST23 isolates identical according to the core genome analysis were collected at the same day on two different farms, indicating a possible clonal spread in the locality. Both were isolates of yolk sac infection of one-day-old chicken, however, coming from different hatcheries. Colibacillosis outbreaks caused by a specific pathogenic clone have been repeatedly reported (e.g. [12, 34]). On the other hand, a closely related isolate (25 SNPs difference) had been collected on an unrelated farm approximately half a year before. Similar situation was observed in the ST429 cluster – the most similar isolates were from the same date and were separated from the other isolates of this cluster (with 26–61 SNPs difference) by a span of several months. One may speculate these isolates could have a common origin, however, the question, whether these clones may become established somehow in the production chain and circulate between flocks or farms for a long time period or whether a repeated introduction occurs from a specific source, remains unanswered. The isolates of the ST429 cluster were obtained from one-day-old chicken coming from four different hatcheries. Although an evidence for „pseudo-vertical “spread through the production pyramid has been proposed recently [35], this fact suggests that the hatchery is probably not the source. The problem of possible reservoir of pathogenic strains for Northern Moravian farms should be addressed more closely in the future.

Both ST429 and ST23 are considered as predominant APEC lineages that are frequently isolated from poultry with clinically manifested disease [34, 36], but also poultry products [25]. Although representing quite unrelated APEC clades, they both appear to be poultry-specific, with little pathogenic potential for humans [7, 16]. In fact, an APEC strain χ7122 (ST23) has been shown to be phylogenetically closer to human ETEC (without any enterotoxin production) than to ExPEC [37]. Therefore, in our collection, one may consider the two clusters, ST429 and ST23, representatives of phylogenetically distant lineages presumably associated with the same disease, again underlining the importance of accessory genome in virulence potential of APEC. The ST429 isolates had a slightly higher average number of virulence-asociated genes than ST23 isolates (172 vs. 154) including genes encoding capsule production (*kpsM, T, D, neuC*), invasins (*ibeA, ompA*) and iron-binding systems (aerobactin, yersiniabactin, *chu*) that the ST23 cluster (not all ST23 isolates) lacked. In contrast, ST23 isolates were characterized by presence of Stg fimbriae and ETT2-related genes. This transport system, even in degenerate state, has been reported to enhance virulence in APEC [38]. Both sequence types coded for curli, F1 fimbriae, salmochelin, OmpT, TraT, Iss, however, only Iro, OmpT nad Iss have been reported to occur in significantly higher prevalence in APEC than avian-faecal *E. coli* (AFEC) [4]. Nevertheless, it probably supports the idea of feasibility and usefulness of PCR typing targeting such potential markers of APEC derived from distant phylogenetic groups (e.g. [3]).

Two isolates were assigned to ST117 (phylogenetic group F). Recent studies indicate that this sequence type comprises important APEC lineages that are repeatedly reported from colibacillosis outbreaks in different countries [24, 36, 39–41], but are also highlighted as potential zoonotic pathogens for containing ExPEC-related virulence genes and being isolated from both retail poultry meat and human clinical urinary tract infections [42]. The remaining phylogroup F isolate was ST354, another potentially zoonotic ST, reported particularly from human and animal healthcare facilities and characterized by common resistance to antimicrobials including fluoroquinolones [43, 44]. This isolate carried *bla*_CMY-2,-59_ and encoded multiple adhesins including K99/F5 fimbriae, which were not found anywhere else. Both ST117 and ST354 were highly prevalent among ESBL/AmpC positive chicken isolates and it has been proposed that these lineages exhibit particularly effective host colonization and persistence in the environment [40, 44].

ST95 is probably the most important pandemic ExPEC lineage that is frequently isolated from chickens [22, 24, 45]. In fact, it may represent, along with closely related ST140, that part of B2 phylogroup where human ExPEC and APEC form a single „subpathotype “of genetically indistingushable strains [16–18]. In humans, ST95 was associated with bloodstream infections, UTIs and meningitis, often characterized by serogroups O1, O2, O45, flagellar antigen H7 and K1 capsule (typical feature of NMEC) and, in contrast to other pandemic lineages, relatively low tendency to acquire antimicrobial resistence [19, 46]. Indeed, not every ST95 seems to be zoonotic, as was shown with APEC O1 in a murine infectious model [47]. On the other hand, our ST95 isolate fulfilled the molecular criteria for UPEC as defined by Johnson et al. [48].

Antimicrobial-resistance profile ranged from full susceptibility to all antimicrobials tested to multidrug resistance, with dominating resistance to β-lactams (ampicillin) and first generation quinolones (nalidixic acid). Resistance to β-lactams was associated largely with TEM-type β-lactamase production. No selection procedure to obtain ESBL/AmpC producing isolates had been used and we did not detect any *bla*_CTX-M_ gene, while four isolates carried *bla*_CMY-2_ gene. This gene, along with *bla*_CTX-M-1_, is the most common ESBL/AmpC β-lactamase in poultry *E. coli* isolates [49]. While in most quinolone-resistant isolates a chromosomal mutation in *gyrA* gene was detected, seven both susceptible or resistant isolates carried *qnrS-1*. Co-occurence of *bla-*_CMY-2, − 59_ and *qnrS-1* was observed in two isolates from the ST429 cluster and all but one ST23 isolates carried the remaining *qnrS-1* genes. One may assume that the afore-mentioned fact that these STs are not commonly associated with human disease does not make them epidemiologically irrelevant, for they may still serve as a source of resistance or virulence determinants in horizontal gene transfer. Indeed, the importance of horizontal gene transfer may be assumed from the detection of a multitude of replicons previously associated with both resistance and virulence gene spread [50–54].

## Conclusions

Despite its limitations due to relatively small number of isolates of completely sequenced isolates, this study could be considered a basic overview of the diversity of colibacillosis-associated *E. coli* isolates and a delineation of paths that are to be followed in more extensive monitoring of virulent clones occuring in central Europe, as well as more elaborate analysis of their phylogenetic background and accessory genome and the role they play in adaptation of different APEC lineages to different hosts, infection types and routes of transmission. Genomic analysis of a collection of poultry colibacillosis-associated isolates revealed two clusters of phylogenetically distant lineages (ST429 and ST23) alongside a great diversity of other sequence types. In general, the collection showed a split into isolates from phylogroups F, B2 and D on one side and A, C and B1 on the other, distinctly differing in distribution of several virulence-associated genes. Clearly more research is needed to assess whether they differ also in their virulence potential and other features.

## Methods

### Strains isolation and preliminary characterisation

Samples have been collected since 2014 at various Czech, Slovakian and Romanian farms with increased mortality due to colibacillosis: mostly internal organ swabs from one-day chicken with yolk sac infection and septicemia or from broilers and layers suffering from colisepticemia and polyserositis (peritonitis, perihepatitis, pericarditis, airsaculitis or haemorrhagic septicemia). The origin of isolates is shown in the Table 4 (additional file 2). The chicken, from which the sequenced isolates were obtained, originated from 4 different hatcheries in the Czech Republic, Slovakia and Hungary. For 3 isolates, the hatchery has not been traced back. For the codes indicating the farm and hatchery of origin please see Table 4 (additional file 2). The samples were cultivated on McConkey agar (37 °C for 18 h aerobically) (Oxoid, UK), subcultivated on Columbia blood agar (the same conditions) (Oxoid, UK) and identified by MALDI-TOF MS (Bruker-Daltonics, Germany). Antibiotic susceptibility to selected antimicrobials – ampicillin (10 μg), amoxicillin-clavulanic acid (20/10 μg), cephalotin (30 μg), sulphonamide compounds (250–300 μg), gentamicin (10 μg), nalidixic acid (30 μg), sulphamethoxasol-trimethoprim (1,25/23,75 μg), tetracycline (30 μg), chloramphenicol (30 μg), ciprofloxacin (5 μg) – was tested by disc diffusion method (Oxoid, UK) and interpreted according to the Clinical and Laboratory Standard Institute [55].

Within preliminary characterization of 95 isolates, a slide agglutination test with four commercial antisera (O1, O8, O18 and O78) was performed according to the manufacturer’s instructions (Denka Seiken, Japan). Presence of several selected resistance and virulence-associated genes (additional file 3 – table 3) was detected by PCR. After this preliminary characterization 32 strains were selected for whole-genome sequencing. To encompass the greatest possible diversity, we exluded isolates from the same individual, the same farm or the same date of isolation, if they showed the identical resistance phenotype and gene profile.

### DNA extraction and whole-genome sequencing

NucleoSpin Tissue DNA extraction kit (Macherey-Nagel, Germany) following manufacturer’s instructions was used to obtain pure DNA. The DNA libraries were prepared with Nextera XT Library preparation kit (Illumina, USA). Finally, Illumina Next-Seq and Mi-Seq platforms were used for the whole-genome sequencing to obtain 2 × 150-bp or 2 × 300-bp paired-end reads, respectively.

### Data processing

Adaptor residues and low quality (Q ≤ 20) ends were removed from the reads using Trimmomatic v0.36 [56]. De novo assembly was performed using SPAdes assembler v3.12.0 [57]. Contigs were submitted to online typing tools (Centre for Genomic Epidemiology, Technical University of Denmark; http://www.genomicepidemiology.org/): ResFinder 3.1 [58], PlasmidFinder 2.0 [59], SeroTypeFinder 2.0 [60], MLST 2.0 [61]. One isolate was assigned as a novel ST8874 by Enterobase. Presence of resistance and virulence genes was predicted using CARD (Comprehensive Antibiotic Resistance Database) and VFDB (Virulence Factor Database) [62], respectively, in the ABRicate v0.8.13 programme (https://card.mcmaster.ca/, https://github.com/tseemann/abricate). The threshold for gene identity was set to 95%. The Clermont typing tool was used to classify isolates into phylogenetic groups [63] (http://clermontyping.iame-research.center/). In order to investigate the genetic relationships between isolates, genomes were annotated using Prokka v1.13 [64] and the core genome alignment was performed using Roary [65]. The core genome alignment was used to determine the single nucleotide polymorphism (SNP) distance using snp-dist (https://github.com/tseemann/snp-dists). Phylogenetic tree was constructed using RAxML v8.2.10 using GTR + GAMMA+I model [66]. Phylogenetic tree was then visualised via iTOL [67] (https://itol.embl.de/). The raw sequencing data were deposited to GenBank under BioProject PRJNA553636 and corresponding accession numbers to SRA for each sample can be found in Table 4 (additional file 4).

## Supplementary information

**Additional file 1: Table 1.** Origin, date of isolation, serotyping, phylogenetic group, antimicrobial resistance genes and replicon profile of selected isolates. **Figure 1.** Preliminary characterization of 95 isolates – antimicrobial resistance. **Figure 2.** Preliminary characterization of 95 isolates – PCR detection of virulence genes.

**Additional file 2: Table 2.** Overview of virulence-associated genes detected by whole-genome sequencing. **Table 4.** The origin of isolates.

**Additional file 3: Table 3.** Primers used in PCR characterization.

**Additional file 4: Table 4.** Statistics.

## Data Availability

The genome sequences of the 32 isolates are available in the GenBank under the bioproject ID PRJNA553636. The corresponding accession numbers are stated in the Additional file 4. https://www.ncbi.nlm.nih.gov/bioproject/PRJNA553636/.

## Abbreviations

AMR: Antimicrobial resistance
AFEC: Avian fecal *Escherichia coli*
APEC: Avian pathogenic *Escherichia coli*
ESBL: Extended-spectrum beta-lactamases
ETEC: Enterotoxinogenic *Escherichia coli*
ExPEC: Extraintestinal pathogenic *Escherichia coli*
MLST: Multilocus sequence typing
NMEC: Neonatal meningitis *Escherichia coli*
PCR: Polymerase chain reaction
PMQR: Plasmid-mediated quinolone resistance
SePEC: Septicemia-associated pathogenic *Escherichia coli*
SHS: Swollen head syndrome
SNP: Single nucleotide polymorphism
ST: Sequence type
UPEC: Uropathogenic *Escherichia coli*
UTI: Urinary tract infection
WHS: Whole genome sequencing

## References

[1] Nolan LK, Barnes HJ, Vaillancourt JP, Abdul-Aziz T, Logue CM, Swayne DE, Glisson JR (2013). Colibacillosis. Diseases of poultry.

[2] La Ragione RM, Woodward MJ (2002). Virulence factors of Escherichia coli serotypes associated with avian colisepticaemia. Res Vet Sci.

[3] Johnson TJ, Wannemuehler Y, Doetkott C, Johnson SJ, Rosenberger SC, Nolan LK (2008). Identification of minimal predictors of avian pathogenic *Escherichia coli* virulence for use as a rapid diagnostic tool. J Clin Microbiol.

[4] Rodriguez-Siek KE, Giddings CW, Doetkott C, Johnson TJ, Nolan LK (2005). Characterizing the APEC pathotype. Vet Res.

[5] Stromberg ZR, Johnson JR, Fairbrother JM, Kilbourne J, Van Goor A, Curtiss R, Mellata M (2017). Evaluation of *Escherichia coli* isolates from healthy chickens to determine their potential risk to poultry and human health. PLoS One.

[6] Collingwood C, Kemmett K, Williams N, Wigley P (2014). Is the concept of avian pathogenic Escherichia coli as a single pathotype fundamentally flawed?. Front Vet Sci.

[7] Ewers C, Antão EM, Diehl I, Philipp HC, Wieler LH (2009). Intestine and environment of the chicken as reservoirs for extraintestinal pathogenic *Escherichia coli* strains with zoonotic potential. Appl Environ Microbiol.

[8] Kemmett K, Humphrey T, Rushton S, Close A, Wigley P, Williams NJ (2013). A longitudinal study simultaneously exploring the carriage of APEC virulence associated genes and the molecular epidemiology of faecal and systemic *E. coli* in commercial broiler chickens. PLoS One.

[9] Maturana VG, de Pace F, Carlos C, Mistretta Pires M, Amabile de Campos T, Nakazato G, Guedes Stheling E, Logue CM, Nolan LK, Dias da Silveira (2011). Subpathotypes of avian pathogenic *Escherichia coli* (APEC) exist as defined by their syndromes and virulence traits. Open Microbiol J.

[10] Jordan FTW, Williams NJ, Wattret A, Jones T (2005). Observations on salpingitis, peritonitis and salpingoperitonitis in a layer breeder flock. Vet Rec.

[11] Olsen RH, Thøfner ICN, Pors SE, Pires dos Santos T, Christensen JP (2016). Experimental induced avian E coli salpingitis: significant impact of strain and host factors on the clinical and pathological outcome. Vet Microbiol.

[12] Pires-dos-Santos T, Bisgaard M, Christensen H (2013). Genetic diversity and virulence profiles of *Escherichia coli* causing salpingitis and peritonitis in broiler breeders. Vet Microbiol.

[13] Mellata M (2013). Human and avian extraintestinal pathogenic *Escherichia coli*: infections, zoonotic risks, and antibiotic resistance trends. Foodborne Pathog Dis.

[14] Johnson JR, Russo TA (2002). Extraintestinal pathogenic *Escherichia coli*: “The other bad E coli”. J Lab Clin Med.

[15] Guabiraba R, Schouler C (2015). Avian colibacillosis: still many black holes. FEMS Microbiol Lett.

[16] Zhu Ge X, Jiang J, Pan Z, Hu L, Wang S, Wang H, Leung FC, Dai J, Fan H (2014). Comparative genomic analysis shows that avian pathogenic *Escherichia coli* isolate IMT5155 (O2:K1:H5; ST complex 95, ST140) shares close relationship with ST95 APEC O1:K1 and human ExPEC O18:K1 strains. PLoS One.

[17] Moulin-Schouleur M, Répérant M, Laurent S, Brée A, Mignon-Grasteau S, Germon P, Rasschaert D, Schouler C (2007). Extraintestinal pathogenic *Escherichia coli* strains of avian and human origin: link between phylogenetic relationships and common virulence patterns. J Clin Microbiol.

[18] Tivendale KA, Logue CM, Kariyawasam S, Jordan D, Hussein A, Li G, Wannemuehler Y, Nolan LK (2010). Avian-pathogenic Escherichia coli strains are similar to neonatal meningitis *E. coli* strains and are able to cause meningitis in the rat model of human disease. Infect Immun.

[19] Riley LW (2014). Pandemic lineages of extraintestinal pathogenic *Escherichia coli*. Clin Microbiol Infect.

[20] Cunha MPV, Saidenberg AB, Moreno AM, Ferreira AJP, Vieira MAM, Gomes TAT, Knöbl T (2017). Pandemic extra-intestinal pathogenic *Escherichia coli* (ExPEC) clonal group O6-B2-ST73 as a cause of avian colibacillosis in Brazil. PLoS One.

[21] Liu CM, Stegger M, Aziz M, Johnson TJ, Waits K, Nordstrom L, Gauld L, Weaver B, Rolland D, Statham S, Horwinski J, Sariya S, Davis GS, Sokurenko E, Keim P, Johnson JR, Price LB (2018). *Escherichia coli* ST131-H22 as a foodborne uropathogen. mBio.

[22] Maluta RP, Logue CM, Casas MRT, Meng T, Guastalli EAL, Rojas TCG, Montelli AC, Sadatsune T, de Carvalho Ramos M, Nolan LK, da Silveira WD (2014). Overlapped sequence types (STs) and serogroups of avian pathogenic (APEC) and human extra-intestinal pathogenic (ExPEC) *Escherichia coli* isolated in Brazil. PLoS One.

[23] Manges AR (2016). *Escherichia coli* and urinary tract infections: the role of poultry-meat. J Clin Microbiol Infect.

[24] Mora A, Viso S, López C, Alonso MP, García-Garrote F, Dabhi G, Mamani R, Herrera A, Marzoa J, Blanco M, Blanco JE, Moulin-Schouleur M, Schouler C, Blanco J (2013). Poultry as reservoir for extraintestinal pathogenic *Escherichia coli* O45:K1:H7-B2-ST95 in humans. Vet Microbiol.

[25] Pietsch M, Irrgang A, Roschanski N, Brenner Michael G, Hamprecht A, Rieber H, Käsbohrer A, Schwarz S, Rösler U, Kreienbrock L, Pfeifer Y, Fuchs S, Werner G, RESET Study Group (2018). Whole genome analyses of CMY-2-producing *Escherichia coli* isolates from humans, animals and food in Germany. BMC Genomics.

[26] Cordoni G, Woodward MJ, Wu H, Alanazi M, Wallis T, La Ragione RM (2016). Comparative genomics of European avian pathogenic *E. coli* (APEC). BMC Genomics.

[27] Paudel S, Stessl B, Hess C, Zloch A, Hess M (2016). High genetic diversity among extraintestinal Escherichia coli isolates in pullets and layers revealed by a longitudinal study. BMC Vet Res.

[28] Clermont O, Christenson JK, Denamur E, Gordon DM (2013). The Clermont *Escherichia coli* phylo-typing method revisited: improvement of specificity and detection of new phylo-groups. Environ Microbiol Rep.

[29] Johnson TJ, Siek KE, Johnson SJ, Nolan LK (2006). DNA sequence of a ColV plasmid and prevalence of selected plasmid-encoded virulence genes among avian *Escherichia coli* strains. J Bacteriol.

[30] Mellata M, Dho-Moulin M, Dozois CM, Curtiss R, Brown PK, Arné P, Brée A, Desautels C, Fairbrother JM (2003). Role of virulence factors in resistance of avian pathogenic Escherichia coli to serum and in pathogenicity. Infect Immun.

[31] Tenaillon O, Skurnik D, Picard B, Denamur E (2010). The population genetics of commensal *Escherichia coli*. Nat Rev Microbiol.

[32] Mokady D, Gophna U, Ron EZ (2005). Extensive gene diversity in septicemic Escherichia coli strains. J Clin Microbiol.

[33] Escobar-Parámo P, Blanc-Potard AB, Bui H, Le Bouguénec C, Denamur E (2004). A specific genetic background is required for acquisition and expression of virulence factors in *Escherichia coli*. Mol Biol Evol.

[34] Solà-Ginés M, Cameron-Veas K, Badiola I, Dolz R, Majó N, Dahbi G, Viso S, Mora A, Blanco J, Piedra-Carrasco N, González-López JJ, Migura-Garcia L (2015). Diversity of multi-drug resistant avian pathogenic *Escherichia coli* (APEC) causing outbreaks of colibacillosis in broilers during 2012 in Spain. PLoS One.

[35] Projahn M, Daehre K, Roesler U, Friese A (2016). Extended-spectrum-beta-lactamase- and plasmid-encoded cephamycinase-producing enterobacteria in the broiler hatchery as a potential mode of pseudo-vertical transmission. J Appl Environ Microbiol.

[36] Cummins ML, Reid CJ, Roy Chowdhury P, Bushell RN, Esbert N, Tivendale KA, Noormohammadi AH, Islam S, Marenda MS, Browning GF, Markham PF, Djordjevic SP (2019). Whole genome sequence analysis of Australian avian pathogenic *Escherichia coli* that carry the class 1 integrase gene. Microb Genom.

[37] Dziva F, Hauser H, Connor TR, van Diemen PM, Prescott G, Langridge GC, Eckert S, Chaudhuri RR, Ewers C, Mellata M, Mukhopadhyay S, Curtiss R, Dougan G, Wieler LH, Thomson NR, Pickard DJ, Stevens MP (2013). Sequencing and functional annotation of avian pathogenic *Escherichia coli* serogroup O78 strains reveal the evolution of *E. coli* lineages pathogenic for poultry via distinct mechanisms. Infect Immun.

[38] Ideses D, Gophna U, Paitan Y, Chaudhuri RR, Pallen MJ, Ron EZ (2005). A degenerate type III secretion system from septicemic *Escherichia coli* contributes to pathogenesis. J Bacteriol.

[39] Dissanayake DRA, Octavia S, Lan R (2014). Population structure and virulence content of avian pathogenic *Escherichia coli* isolated from outbreaks in Sri Lanka. Vet Microbiol.

[40] Projahn M, Daehre K, Semmler T, Guenther S, Roesler U, Friese A (2018). Environmental adaptation and vertical dissemination of ESBL−/pAmpC-producing *Escherichia coli* in an integrated broiler production chain in the absence of an antibiotic treatment. Microb Biotechnol.

[41] Ronco T, Stegger M, Olsen RH, Sekse C, Nordstoga AB, Pohjanvirta T, Lilje B, Lyhs U, Andersen PS, Pedersen K (2017). Spread of avian pathogenic *Escherichia coli* ST117 O78:H4 in Nordic broiler production. BMC Genomics.

[42] Vincent C, Boerlin P, Daignault D, Dozois CM, Dutil L, Galanakis C, Reid-Smith RJ, Tellier PP, Tellis PA, Ziebell K, Manges AR (2010). Food reservoir for *Escherichia coli* causing urinary tract infections. Emerg Infect Dis.

[43] Guo S, Wakeham D, Brouwers HJM, Cobbold RN, Abraham S, Mollinger JL, Johnson JR, Chapman TA, Gordon DM, Barrs VR, Trott DJ (2015). Human-associated fluoroquinolone-resistant *Escherichia coli* clonal lineages, including ST354, isolated from canine feces and extraintestinal infections in Australia. Microbes Infect.

[44] Vangchhia B, Abraham S, Bell JM, Collignon P, Gibson JS, Ingram PR, Johnson JR, Kennedy K, Trott DJ, Turnidge JD, Gordon DM (2016). Phylogenetic diversity, antimicrobial susceptibility and virulence characteristics of phylogroup F *Escherichia coli* in Australia. Microbiology.

[45] Jørgensen SL, Stegger M, Kudirkiene E, Lilje B, Poulsen LL, Ronco T, Pires Dos Santos T, Kiil K, Bisgaard M, Pedersen K, Nolan LK, Price LB, Olsen RH, Andersen PS, Christensen H (2019). Diversity and population overlap between avian and human *Escherichia coli* belonging to sequence type 95. mSphere.

[46] Mora A, López C, Dabhi G, Blanco M, Blanco JE, Alonso MP, Herrera A, Mamani R, Bonacorsi S, Moulin-Schouleur M, Blanco J (2009). Extraintestinal pathogenic *Escherichia coli* O1:K1:H7/NM from human and avian origin: detection of clonal groups B2 ST95 and D ST59 with different host distribution. BMC Microbiol.

[47] Johnson TJ, Wannemuehler Y, Kariyawasam S, Johnson JR, Logue CM, Nolan LK (2012). Prevalence of avian-pathogenic *Escherichia coli* strain O1 genomic islands among extraintestinal and commensal *E. coli* isolates. J Bacteriol.

[48] Johnson JR, Murray AC, Gajewski A, Sullivan M, Snippes P, Kuskowski MA, Smith KE (2003). Isolation and molecular characterization of nalidixic acid-resistant extraintestinal pathogenic *Escherichia coli* from retail chicken products. Antimicrob Agents Chemother.

[49] van Hoek AHAM, Stalenhoef JE, van Duijkeren E, Franz E (2016). Comparative virulotyping of extended-spectrum cephalosporin-resistant *E. coli* isolated from broilers, humans on broiler farms and in the general population and UTI patients. Vet Microbiol.

[50] Dolejska M, Villa L, Poirel L, Nordmann P, Carattoli A (2013). Complete sequencing of an IncHI1 plasmid encoding the carbapenemase NDM-1, the ArmA 16S RNA methylase and a resistance–nodulation–cell division/multidrug efflux pump. J Antimicrob Chemother.

[51] Dolejska M, Duskova E, Rybarikova J, Janoszowska D, Roubalova E, Dibdakova K, Maceckova G, Kohoutova L, Literak I, Smola J, Cizek A (2011). Plasmids carrying blaCTX-M^−1^ and qnr genes in *Escherichia coli* isolates from an equine clinic and a horseback riding centre. J Antimicrob Chemother.

[52] Johnson TJ, Nolan LK (2009). Pathogenomics of the virulence plasmids of *Escherichia coli*. Microbiol Mol Biol Rev.

[53] Kao CY, Chen JW, Liu TL, Yan JJ, Wu JJ (2018). Comparative genomics of *Escherichia coli* sequence type 219 clones from the same patient: evolution of the IncI1 bla_CMY_-carrying plasmid in vivo. Front Microbiol.

[54] Royden A, Ormandy E, Pinchbeck G, Pascoe B, Hitchings MD, Sheppard SK, Williams NJ (2019). Prevalence of faecal carriage of extended-spectrum β-lactamase (ESBL)-producing *Escherichia coli* in veterinary hospital staff and students. Vet Rec Open.

[55] Clinical and Laboratory Standards Institute (CLSI) (2015). Performance standards for antimicrobial susceptibility testing; twenty-fifth informational supplement. CLSI document M100-S25.

[56] Bolger AM, Lohse M, Usadel B (2014). Trimmomatic: a flexible trimmer for Illumina sequence data. Bioinformatics.

[57] Bankevich A, Nurk S, Antipov D, Gurevich AA, Dvorkin M, Kulikov AS, Lesin VM, Nikolenko SI, Pham S, Prjibelski AD, Pyshkin AV, Sirotkin AV, Vyahhi N, Tesler G, Alekseyev MA, Pevzner PA (2012). SPAdes: a new genome assembly algorithm and its applications to single-cell sequencing. J Comput Biol.

[58] Zankari E, Hasman H, Cosentino S, Vestergaard M, Rasmussen S, Lund O, Aarestrup FM, Larsen MV (2012). Identification of acquired antimicrobial resistance genes. J Antimicrob Chemother.

[59] Carattoli A, Zankari E, García-Fernández A, Voldby Larsen M, Lund O, Villa L, Møller Aarestrup F, Hasman H (2014). In silico detection and typing of plasmids using PlasmidFinder and plasmid multilocus sequence typing. Antimicrob Agents Chemother.

[60] Joensen KG, Tetzschner AM, Iguchi A, Aarestrup FM, Scheutz F (2015). Rapid and easy in silico serotyping of *Escherichia coli* using whole genome sequencing (WGS) data. J Clin Microbiol.

[61] Larsen MV, Cosentino S, Rasmussen S, Friis C, Hasman H, Marvig RL, Jelsbak L, Sicheritz-Pontén T, Ussery DW, Aarestrup FM, Lund O (2012). Multilocus sequence typing of total-genome-sequenced bacteria. J Clin Microbiol.

[62] Liu B, Zheng D, Jin Q, Chen L, Yang J (2019). VFDB 2019: a comparative pathogenomic platform with an interactive web interface. Nucleic Acids Res.

[63] Beghain J, Bridier-Nahmias A, Le Nagard H, Denamur E, Clermont O (2018). ClermonTyping: an easy-to-use and accurate in silico method for *Escherichia* genus strain phylotyping. Microb Genom.

[64] Seemann T (2014). Prokka: rapid prokaryotic genome annotation. Bioinformatics.

[65] Page JA, Cummins CA, Hunt M, Wong VK, Reuter S, Holden MTG, Fookes M, Falush D, Keane JA, Parkhill J (2015). Roary: rapid large-scale prokaryote pan genome analysis. Bioinformatics.

[66] Stamatakis A (2014). RAxML version 8: a tool for phylogenetic analysis and post-analysis of large phylogenies. Bioinformatics.

[67] Letunic I, Bork P (2016). Interactive tree of life (iTOL) v3: an online tool for the display and annotation of phylogenetic and other trees. Nucleic Acids Res.

