# Supplementary material for: Genomic analysis of Escherichia coli strains isolated from diseased chicken in the Czech Republic
Source: BMC Vet Res. 2020 Jun 10;16:189. doi: 10.1186/s12917-020-02407-2 (PMC7286222; doi:10.1186/s12917-020-02407-2)
Supplement: Supplementary file 1 — Additional file 1: Table 1. Origin, date of isolation, serotyping, phylogenetic group, antimicrobial resistance genes and replicon profile of selected isolates. Figure 1. Preliminary characterization of 95 isolates – antimicrobial resistance. Figure 2. Preliminary characterization of 95 isolates – PCR detection of virulence genes. [file 12917_2020_2407_MOESM1_ESM.docx]

| Isolate | Syndrome | | Date | Serotype | Phylo- group | MLST | AMR genes | Replicon |
| --- | --- | --- | --- | --- | --- | --- | --- | --- |
| 587 | PS | 7.3.2016 | | O78:H4 | F | 117 | *bla*_TEM-1_*, tet(*A*), sul1, sul2, dfrA15, aph(3'')-Ib, aph(6)-Id, aadA* | Col(MG828), ColRNAI, ColpVC, IncFIB, IncFIC |
| 95813 | YSI | 1.9.2014 | | O45:H4 | F | 117 | *tet(*B*), sul2, aph(3'')-Ib, aph(3')-Ia, aph(6)-Id* | IncFIA, IncFIB |
| 95815A | YSI | 1.9.2014 | | O17:H10 | B2 | 8874* | not found | Col(MG828), IncB/O/K/Z_2, IncFIB, IncX1 |
| 96079 | YSI | 25.9. 2014 | | NT:H5 | B2 | 352 | not found | ColRNAI, IncFIB, IncFII |
| 95917 | YSI | 14.9. 2014 | | O1:H7 | B2 | 95 | not found | IncFIB, IncI1 |
| 2319 | PS | 21.9. 2016 | | O2-O50:H5 | B2 | 140 | *bla*_TEM-1_*, sul2, aph(3'')-Ib, aph(*6*)-Id, catA1* | IncB/O/K/Z_2, IncFIB, IncFIC |
| 96012 | YSI | 8.9.2014 | | NT:H4 | B2 | 4110 | not found | Col(MG828), IncFIB, IncX1 |
| 100655 | PS | 3.12. 2015 | | O2:H1 | B2 | 429 | *bla*_CMY-2_*, bla*_CMY-59_*, tet(*A,C*), sul1, aac(3)-Via, aadA* | Col(MG828), ColRNAI, ColpVC, IncFIB, IncI1, IncQ1 |
| 96099 | YSI | 7.10. 2014 | | NT:H1 | B2 | 429 | not found | ColRNAI, IncFIB, pHI1B, p0111 |
| 96080 | YSI | 25.9. 2014 | | NT:H1 | B2 | 429 | *bla*_TEM-106_*, bla*_TEM-135_, *bla*_CMY-2_*, bla*_CMY-59_*, qnrS1, tet(*A*), sul1, aac(3)-VIa, aadA* | Col(MG828), Col156, ColRNAI, ColpVC, IncB/O/K/Z_4, IncFIB, IncFII, p0111 |
| 96093C | YSI | 7.10. 2014 | | O8:H9 | B2 | 429 | *bla*_TEM-106_*, bla*_TEM-135_, *bla*_CMY-2_*, bla*_CMY-59,_ *qnrS1, tet(*A*), sul1, aadA* | IncFIB |
| 96098C | YSI | 7.10. 2014 | | NT:H34 | F | 354 | *bla*_CMY-2_*, bla*_CMY-59,_ *tet(A), sul2, aph(3'')-Ib, aph(3')-Ia,, aph(6)-Id, catA1* | IncB/O/K/Z_2, IncFIB, IncFIC |
| 1102 | PS | 26.4.2016 | | O15:H6 | D | 1249 | *tet(*B*)* | ColRNAI, IncFIB, IncFIC |
| 96094 | YSI | 7.10. 2014 | | NT:H2 | D | 1914 | *bla*_TEM-1_*, dfrA5* | Col(MG828), ColRNAI, IncFIB, IncFII |
| 95810 | YSI | 1.9.2014 | | O15:H16 | clade I | 770 | *tet(*A*), sul1, aac(3)-VIa, aadA* | Col(MG828), ColRNAI, IncI1, IncY |
| 95824 | YSI | 1.9.2014 | | O5:H10 | A | 93 | not found | Col(MG828), ColRNAI, IncFIB, IncQ1 |
| 2326 | PS | 21.9. 2016 | | O75:H42 | A | 2223 | *bla*_TEM-1_*, sul2, dfrA15* | Col(MG828), ColRNAI, IncFIB |
| 96092 | YSI | 7.10. 2014 | | O147:H6 | A | 10 | *bla*_TEM-1_ | ColRNAI, IncFIB, IncFIC |
| 96096 | YSI | 7.10. 2014 | | NT:H18 | A | 746 | *bla*_TEM-1_ | Col(MG828), ColRNAI, IncB/O/K/Z_2, IncFIC, IncX1 |
| 96094C | YSI | 7.10. 2014 | | O8:H9 | C | 23 | *qnrS1* | Col(MG828), ColRNAI, IncFIB, IncFII, p0111 |
| 96006 | YSI | 8.9.2014 | | O78:H9 | C | 23 | *bla*_TEM-1_*, sul2, dfrA14, aph(3'')-Ib, aph(6)-Id* | Col(MG828), Col156, IncFIB |
| 96097C | YSI | 7.10. 2014 | | O8:H9 | C | 23 | *bla*_TEM-106_*, bla*_TEM-135_*, qnrS1, tet(A)* | Col(MG828), ColpVC, IncB/O/K/Z_2, IncFIB, IncX1 |
| 96099R | YSI | 7.10. 2014 | | O8:H9 | C | 23 | *bla*_TEM-106_*, bla*_TEM-135_*, qnrS1, tet(A)* | IncHI1 |
| 96095 | YSI | 7.10. 2014 | | O8:H9 | C | 23 | *bla*_TEM-106_*, bla*_TEM-135_*, qnrS1, tet(*A*)* | Col(MG828), Col156, ColRNAI, ColpVC, IncB/O/K/Z_4, IncFIB, IncQ1 |
| 95805C | YSI | 1.9.2014 | | O8:H9 | C | 23 | *bla*_TEM-106_*, bla*_TEM-135_*, qnrS1* | IncFIB, IncFIC |
| 1101 | PS | 26.4. 2016 | | O20:H14 | B1 | 1249 | not found | IncFIB |
| 95802 | YSI | 1.9.2014 | | O8:H19 | B1 | 162 | not found | IncFIB |
| 2327 | PS | 21.9. 2016 | | O91:H27 | B1 | 1157 | *bla*_TEM-1_*, tet(*A*), sul1, sul2, dfrA15, aph(3'')-Ib, aadA* | ColRNAI, IncFIB, IncFIC |
| 96093 | YSI | 7.10. 2014 | | O4:H21 | B1 | 602 | not found | IncFIB, IncFIC |
| 2325 | PS | 21.9. 2016 | | O103:H7 | B1 | 1841 | *bla*_TEM-1_*, tet(*A*)* | Col(MG828), IncB/O/K/Z_4, IncFIB, IncFIC, IncX1 |
| 1104 | PS | 26.4. 2016 | | O76:H14 | B1 | 533 | *bla*_TEM-30_*, tet(*A*), sul1, sul2, dfrA15,ant(2'')-Ia, floR* | Col(MG828), IncB/O/K/Z_2, IncFIB |
| 96081 | YSI | 25.9. 2014 | | NT:H16 | B1 | 7104 | not found | Col(MG828), ColpVC, IncB/O/K/Z_2, IncFIB, IncX1 |

Table 1 Origin, date of isolation, serotyping, phylogenetic group, antimicrobial resistance genes and replicon profile of selected isolates

*novel sequence type (Enterobase)

PS – polyserositis; YSI – yolk sac infection

Figure 1 Preliminary characterization of 95 isolates – antimicrobial resistance

AMP – ampicilin; AMC – amoxicillin-clavulanic acid; KF – cephalotin; S3- sulphonamide compound; SXT – sulphamethoxazol-trimethoprim; TET – tetracycline; C – chloramphenicol; CN – gentamicin; NA – nalidixic acid; CIP – ciprofloxacin

Figure 2 Preliminary characterization of 95 isolates – PCR detection of virulence genes
