# Supplementary material for: Genomic analysis of Escherichia coli strains isolated from diseased chicken in the Czech Republic
Source: BMC Vet Res. 2020 Jun 10;16:189. doi: 10.1186/s12917-020-02407-2 (PMC7286222; doi:10.1186/s12917-020-02407-2)
Supplement: Supplementary file 3 — Additional file 3: Table 3. Primers used in PCR characterization. [file 12917_2020_2407_MOESM3_ESM.docx]

Table 3 Primers used in PCR characterization

| Primer | Sequence | Gene |  | Product (bp) | Reference |
| --- | --- | --- | --- | --- | --- |
| blaTEM-F | ATT CTT GAA GAC GAA AGG GC | *bla*_TEM_ |  | 1150 | Brinas et al., 2002  Ng et al., 1999 |
| blaTEM-R | ACG CTC AGT GGA ACG AAA AC |  |  |  |  |
| blaSHV-F | CAC TCA AGG ATG TAT TGT G | *bla*_SHV_ |  | 885 |  |
| blaSHV-R | TTA GCG TTG CCA GTG CTC G |  |  |  |  |
| tetA/F | GCT ACA TCC TGC TTG CCT TC | *tetA* |  | 210 | Ng et al., 1999  Ng et al., 1999    Ng et al., 1999 |
| tetA/R | CAT AGA TCG CCG TGA AGA GG |  |  |  |  |
| tetB/F | TTG GTT AGG GGC AAG TTT TG | *tetB* |  | 659 |  |
| tetB/R | GTA ATG GGC CAA TAA CAC CG |  |  |  |  |
| tetD/F | AAA CCA TTA CGG CAT TCT GC | *tetD* |  | 787 |  |
| tetD/R | GAC CGG ATA CAC CAT CCA TC |  |  |  |  |
| sul1/F | CTT CGA TGA GAG CCG GCG GC | *sul1* |  | 417 | Zhao et al., 2001 |
| sul1/R | GCA AGG CGG AAA CCC GCG CC |  |  |  |  |
| sul2/F | AGG GGG CAG ATG TGA TCG AC | *sul2* |  | 249 | Faldynova et al., 2003 |
| sul2/R | GCA GAT GAT TTC GCC AAT TG |  |  |  |  |
| sul3/F | GAG CAA GAT TTT TGG AAT CG | *sul3* |  | 789 | Perreten et al., 2003 |
| sul3/R | CAT CTG CAG CTA ACC TAG GGC TTT GGA |  |  |  |  |
| ompT/F | TTG cta ctg cac tct cag c | *ompT* |  | 610 | Bethe et al, 2012 |
| ompT/R | CGA CAG ATA CTC TGG GTA ACA |  |  |  |  |
| iroN/F | ATC CTC TGG TCG CTA ACT G | *iroN* |  | 847 | Ewers et al., 2007  Ewers et al., 2007 |
| iroN/R | CTG CAC TGG AAG AAC TGT TCT |  |  |  |  |
| iss/F | ATC ACA TAG GAT TCT GCC G | *iss* |  | 309 |  |
| iss/R | CAG CGG AGT ATA GAT GCC A |  |  |  |  |
| cvaC/F | CAC ACA CAA ACG GGA GCT GTT | *cvaC* |  | 679 | Dissanayake et al., 2008 |
| cvaC/R | CTT CCC GCA GCA TAG TTC CAT |  |  |  |  |
| tsh/F | GGT GGT GCA CTG GAG TGG | *tsh* |  | 620 | Moulin-Schouler et al., 2007 |
| tsh/R | AGT CCA GCG TGA TAG TGG |  |  |  |  |
| iutA/F | GGC TGG ACA TCA TGG GAA CTG G | *iutA* |  | 300 | Johnson et Stell, 2000 |
| iutA/R | CGT CGG GAA CGG GTA GAA TCG |  |  |  |  |
| frz/F | TCA GTA AGA ACG AAA GTG TG | *frz_orf4_* |  | 565 | Dissanayake et al., 2014 |
| frz/R | ACA GGA ACA ATC CCG TGG AT |  |  |  |  |
| felA/F | GGT CAA SCA GCT AAA AAC GGT AAG G | *felA* |  | 239 | Moulin-Schouler et al., 2006 |
| felA/R | CCT TCA GAA ACA GTA CCG CAA TTC G |  |  |  |  |

References

Briñas, L., Zarazaga, M., Sáenz, Y., Ruiz-Larrea, F., Torres, C., 2002. β-Lactamases in Ampicillin-Resistant *Escherichia* *coli* Isolates from Foods, Humans, and Healthy Animals. Antimicrobial Agents and Chemotherapy 46, 3156–3163.

Bethe, A., Janβen, T., Ewers, C., Oelgeschläger, K., Krinke, P., Kűhl, M. 2012. Multiplex PCR I-IX. Institut fűr Mikrobiologie und Tierseuchen, FU Berlin, 16 p.

Dissanayake, D.R.A., Octavia, S., Lan, R., 2014. Population structure and virulence content of avian pathogenic *Escherichia coli* isolated from outbreaks in Sri Lanka. Veterinary Microbiology 168, 403–412.

Ewers, C., Li, G., Wilking, H., Kieβling, S., Alt, K., Antáo, E.-M., Laturnus, C., Diehl, I., Glodde, S., Homeier, T., Böhnke, U., Steinrück, H., Philipp, H.-C., Wieler, L.H., 2007. Avian pathogenic, uropathogenic, and newborn meningitis-causing *Escherichia* *coli*: How closely related are they? International Journal of Medical Microbiology 297, 163–176.

Faldynova, M., Pravcova, M., Sisak, F., Havlickova, H., Kolackova, I., Cizek, A., Karpiskova, R., Rychlik, I., 2003. Evolution of Antibiotic Resistance in *Salmonella enterica* Serovar Typhimurium Strains Isolated in the Czech Republic between 1984 and 2002. Antimicrobial Agents and Chemotherapy 47, 2002–2005.

Johnson, J.R., Stell, A.L., 2000. Extended Virulence Genotypes of *Escherichia coli* Strains from Patients with Urosepsis in Relation to Phylogeny and Host Compromise. The Journal of Infectious Diseases 181, 261–272

Moulin-Schouleur, M., Schouler, C., Tailliez, P., Kao, M.-R., Brée, A., Germon, P., Oswald, E., Mainil, J., Blanco, M., Blanco, J., 2006. Common Virulence Factors and Genetic Relationships between O18:K1:H7 *Escherichia coli* Isolates of Human and Avian Origin. Journal of Clinical Microbiology 44, 3484–3492.

Ng, L.-K., Mulvey, M.R., Martin, I., Peters, G.A., Johnson, W., 1999. Genetic Characterization of Antimicrobial Resistance in Canadian Isolates of Salmonella Serovar Typhimurium DT104. Antimicrobial Agents and Chemotherapy 43, 3018–3021.

Perreten, V., Boerlin, P., 2003. A New Sulfonamide Resistance Gene (*sul3*) in *Escherichia* *coli* Is Widespread in the Pig Population of Switzerland. Antimicrobial Agents and Chemotherapy 47, 1169–1172.

Zhao, S., White, D.G., Ge, B., Ayers, S., Friedman, S., English, L., Wagner, D., Gaines, S., Meng, J., 2001. Identification and Characterization of Integron-Mediated Antibiotic Resistance among Shiga Toxin-Producing *Escherichia coli* Isolates. Applied and Environmental Microbiology 67, 1558–1564.
